# Nanoencapsulated Essential Oils for Post-Harvest Preservation of Stored Cereals: A Review

**DOI:** 10.3390/foods13244013

**Published:** 2024-12-12

**Authors:** Akash Maurya, Arati Yadav, Monisha Soni, Kishor Kumar Paul, Umakant Banjare, Manish Kumar Jha, Abhishek Kumar Dwivedy, Nawal Kishore Dubey

**Affiliations:** 1 Laboratory of Herbal Pesticides, Centre of Advanced Study in Botany, Institute of Science, Banaras Hindu University, Varanasi 221005, India; bhuaks20@gmail.com (A.M.); arati@bhu.ac.in (A.Y.); monishasoni1@gmail.com (M.S.); kishorpaul2198@gmail.com (K.K.P.); umakantbanjare@gmail.com (U.B.); mjha1010@gmail.com (M.K.J.); akdwivedy.bot@bhu.ac.in (A.K.D.); 2 Department of Botany, Shri Murli Manohar Town Post Graduate College, Ballia 277001, India

**Keywords:** cereal grains, essential oils, nanoencapsulation, safety, antimicrobial, post-harvest preservation

## Abstract

Cereal grains are frequently attacked by microorganisms and insects during storage and processing, which negatively affects their quality, safety, and market value. Therefore, protecting stored grains from microbial contamination is crucial for food industries, farmers, public health associations, and environmental agencies. Due to the negative impact of synthetic gray chemicals, antimicrobial plant-based essential oils (EOs) can serve as alternative, safer, environmentally friendly preservatives that can prolong the shelf life of cereals. However, high volatility, low solubility, hydrophobicity, and quick oxidation limit their practical applicability. Using nanotechnology for the nanoencapsulation of EOs into polymeric matrices allows sustained release and ensures targeted delivery without significantly altering the organoleptic attributes of cereals, making EOs a new-generation green preservative. This ultimately overcomes the challenges of practical applications. The application of nanoencapsulated EOs in grain storage provides an effective and novel defense against microbes, insects, and other contaminants. Hence, the current review thoroughly examines the preservative potential of nanoencapsulated EOs in terms of antimicrobial and insecticidal efficacy for protecting stored cereal grains. It also highlights the challenges encountered during application and the safety concerns of using nanoencapsulated EOs in protecting cereal grains during post-harvest storage.

## 1. Introduction

Cereal grains are the edible seeds of grasses from the Poaceae family, functioning as a fundamental food source for most of the global population. The most prevalent cereal grains include wheat, rice, maize (corn), barley, oats, rye, sorghum, and millet. Among them, rice, wheat, and corn share 60% of the world’s total grain intake [1]. These grains have a high carbohydrate content and varying concentrations of proteins, dietary fibers, vitamin B, iron, and magnesium [2]. Hence, they are crucial for combating food insecurity around the globe. The cereal grains are often attacked by storage pathogens, such as bacteria like viz. *Campylobacter jejuni*, *Listeria monocytogenes*, *Shigella* spp., *Salmonella* spp., and fungi such as *Fusarium graminearum*, *Aspergillus flavus*, and *Penicillium* spp. The release of toxic metabolites, especially enterotoxins and mycotoxins such as aflatoxin B_1_ (AFB_1_), zearalenone, deoxynivalenol, etc., deteriorate their nutritional and esthetic values [3,4]. Poor storage practices coupled with other environmental factors such as heat, moisture, pH levels, and the presence of bacteria and insects in storehouses are the primary causes of cereal grain contamination by microbe and related mycotoxins [5,6].

To mitigate the effects of bacterial and fungal contamination, various physical and chemical approaches are being employed. Still, the spoilage of cereal grains by microorganisms, particularly due to the presence of resistant spores and thermostable toxins, is a matter of concern. Moreover, due to the adverse effects of synthetic preservatives, consumers are increasingly turning to green alternatives to protect food commodities. This has led to the increased demand for natural antimicrobials in the food industry [7]. Among natural antimicrobials, essential oils (EOs) extracted from plant parts demonstrate remarkable antibacterial, insecticidal, and antioxidant effects [8,9] and with a Generally Recognized as Safe (GRAS) status, they are considered safe for use in food products [10,11].

Based on their chemical makeup, EOs can inhibit oxidative processes, the generation of free radicals, and microbial proliferation in both *in vitro* and food preservation applications, positioning EOs as possible partial or complete alternatives to conventional synthetic preservatives [1,12,13]. Despite this, the primary issues that restrict the widespread use of EOs are excessive volatility, inadequate water solubility, strong fragrance, and susceptibility to oxidation upon exposure to moisture, light, heat, pH, and oxygen [7]. The oxidation of EO constituents is a major restriction on their practical applicability in the food and agricultural sectors. Moreover, the direct application of free EOs affects the organoleptic or sensory properties of preserved food commodities [14,15]. As a result, few EOs have been successfully used as natural antimicrobial agents in the food and agricultural industries in crude form.

To counter these obstacles, an innovative and potentially useful strategy is the nanoencapsulation of EOs, which can benefit the food industry by offering the effective preservation and regulated release of EOs, thereby boosting their efficacy as natural preservatives, flavor enhancers, or functional constituents [16]. The application of nanoencapsulated EOs in the preservation of cereal grains is an emerging area of research in the food industry that can extend the shelf life of cereal grains by utilizing the antimicrobial, antioxidant, and preservative qualities of EOs, while also enhancing their durability, efficacy, and release control [17]. The encapsulation of EOs can be accomplished with a broad variety of encapsulating polymers, including chitosan, cyclodextrin, starch, pullulan, alginate, carrageenans, proteins, and gums [13]. Nanotechnology and nanoencapsulation of EOs have been applied at every step of agricultural production, including the priming of seeds, transportation, storage, and post-harvest administration. In addition, nanoencapsulated EOs have been exploited as active preservation agents in the post-harvest storage of cereal grains. Therefore, the present review focuses on the techniques applied for the encapsulation of EOs and the implementation of nanocapsules in the post-harvest preservation of stored cereal grains, and we discuss the different factors responsible for the post-harvest spoilage of cereal grains, the role of essential oils in protecting stored cereals, the challenges of using essential oils, the benefits of using nanoencapsulated essential oils, the hurdles encountered when using nanoencapsulated essential oils, and future directions.

## 2. Post-Harvest Spoilage of Cereal Grains

Cereal grains are a crucial component of the human diet around the globe and are a major source of energy, carbohydrates, and even vegetable proteins. They include crops like wheat, rice, maize, barley, and oats, which are extensively cultivated for human and livestock consumption [18,19]. The degradation of cereal grains after harvest is a significant issue, especially due to microorganisms and pests, which leads to substantial economic damages and undernutrition. Therefore, it is essential to locate suitable storage facilities for cereal grains once they have been harvested in order to achieve the goals of food security, minimizing economic losses, and preserving the quality of grains to the greatest degree possible [20]. During storage, cereal grains are often attacked by insects, rodents, bacteria, and fungi, all of which negatively impact the quality and safety of stored grains and pose a serious threat to human beings and animals [21]. The major causative agents responsible for the post-harvest spoilage of cereal grains and their management strategies are discussed below.

### 2.1. Insect Infestation

The presence of insect pests in stored cereal grains creates a number of critical issues including the possibility of huge economic losses and concerns regarding food security. Post-harvest insect pests not only lower the quality of processed grains, but they may also cause rancid odors. Coleoptera weevils and lepidoptera stem borers are among the most harmful insects when it comes to agricultural fields and stores for consumers [22]. There are a number of pathogenic insects, which besides transmitting diseases, can lead to the destruction of nutritional properties [23]. The most common insects related to stored cereals include the maize weevil (*Sitophilus zeamais* Motschulsky), rice weevil (*Sitophilus oryzae* L.), granary weevil (*Sitophilus granarius* L.), lesser grain borer (*Rhyzopertha dominica*), flour mill beetle (*Cryptolestes turcicus*), merchant grain beetle (*Oryzaephilus mercator*), rusty grain beetle (*Cryptolestes ferrugineus*), Indian meal moth (*Plodia interpunctella*), red flour beetle (*Tribolium castaneum*), confused flour beetle (*Tribolium confusum*), large flour beetle (*Tribolium destructor* Uyttenboogaar), long-headed flour beetle (*Latheticus oryzae*), yellow mealworm (*Tenebrio molitor* L), Angoumois grain moth (*Sitotroga cerealella*), and saw-toothed grain beetle (*Oryzaephilus surinamensis* L.) [24,25,26]. Recently, Berhe et al. [22] reported that *Rhyzopertha dominica* (lesser grain borer), *Sitotroga cerealella* (Angoumois grain moth), and *Tribolium* spp. (flour beetle) are capable of inflicting significant harm to stored cereal grains. Moreover, infestation can negatively affect seed quality, as findings indicate that the damage caused by insects may reduce germination rates and elevate moisture content, hence promoting the proliferation of mold. Similarly, Forghani and Marouf [27] also investigated insect infestation in stored cereal grains, specifically that associated with pests such as *Sitophilus oryzae* and *Sitophilus granarius*, observing significant damages caused, especially to wheat and barley.

### 2.2. Bacterial Contamination

Bacteria are typically not extensively associated with the deterioration of dry grain owing to storage conditions that are unsuitable for their proliferation. However, it was shown that certain bacterial infections and spore-forming species may endure storage conditions and potentially contaminate processed items. In this context, the presence of bacteria in stored cereal grains poses a significant threat, as it is influenced by the moisture content, the storage conditions, and the microorganisms in the atmosphere. Working out these factors is important in ensuring food safety.

- Moisture Level: High moisture content (more than 30%) in stored barley tends to cause an increase in the growth of microorganisms, especially when the grain is poorly sealed with the appropriate storage materials [28].
- Aerial Microorganisms: During grain handling, the workers are exposed to a high concentration of bacteria and fungi in the air, with levels in some situations exceeding 1 million cubic meters of air [29].
- Specific Contaminants: One study found that 56% of dry food products infected with *Bacillus cereus* including grains is able to survive in storage conditions [30].

Moreover, *Bacillus cereus* represents an important source of contamination because of its ability to form spores and survive in very hostile environments like low-moisture and high-temperature settings. Such spores might undergo a process of dormancy and reactivation during the storage/processing of food, which causes spoilage and the production of most of the enterotoxins leading to food-borne illnesses such as vomiting and diarrhea [31]. Other species, like *Bacillus subtilis* and *Bacillus licheniformis*, are involved in spoilage through the breakdown of carbohydrates, proteins, and fats, thereby reducing the quality and nutritional value of grains [32]. Zavišić et al. [33] suggested that bacteria such as *Lactobacillus* and *Leuconostoc* may be found in cereal grains with high moisture levels. The aforementioned organisms utilize carbohydrates for fermentation, producing lactic acid and other metabolites that lead to souring and undesirable changes in flavor and texture. Additionally, *Pseudomonas fluorescens* is a psychrotrophic bacterium that flourishes at low temperatures, which makes it a big threat in cold storage conditions. Its known ability to break down the proteins and fats contained in grains leads to unwanted taste, rancidity, and ultimately, the deterioration of grain quality [34]. Other species of *Pseudomonas*, like *Pseudomonas putida*, act as saprophytes which form protective films around themselves, making it easy to corrode and rot grains even in harsh environmental conditions [35].

### 2.3. Fungal Contamination

The fungal contamination of cereal grains during storage is a major problem since it can result in the generation of mycotoxins that threaten the health of humans and animals. The degree of contamination is influenced by several factors, including storage conditions and the type of fungus present. Relative humidity is the most important determinant of fungal infection, mostly caused by insufficient grain drying, leading to the formation of hot patches. Several fungal contaminants include *Aspergillus flavus*, *Aspergillus luchuensis*, *Aspergillus niger*, *Aspergillus repens*, *Fusarium oxysporum*, *Rhizopus*, *Mucor*, and *Penicillium*, entering stored grains at relatively high humidity levels (65% to 90%) and reducing the moisture level (14% to 16%) of grains [36]. Storage temperature has a marked effect on the extent and type of microbial spoilage. Low temperature (15 °C) favors *Penicillium* species; room temperature from 20 to 25 °C favors the growth of the *Aspergillus* and *Eurotium* species instead [37]. A significant amount of fungal infections results in the release of mycotoxins, which are toxic secondary metabolites that are hazardous to human health. Presently, there have been over 400 different forms of mycotoxins reported in the literature, where aflatoxin produced by the *Aspergillus flavus* and *Aspergillus parasiticus* is the foremost type, i.e., the most toxic and carcinogenic type, even in low doses, posing serious threats to human health. Thus, the International Agency for Research on Cancer classifies it as a Group I carcinogenic agent [38]. However, strong hepatocarcinogenic mycotoxin AFB_1_ is mostly found in cereals, nuts, grains, and feeds. AFB_1_ and AFB_2_ can be consumed by nursing cattle through contaminated feed, and after that, they can be converted to hydroxylated AFM_1_ and AFM_2_. On the other hand, *Penicillium verrucosum*, a member of the *Penicillium* genus, is known for its ability to contaminate and synthesize the dangerous mycotoxin ochratoxin A (OTA), causing damage to wheat and barley only after harvesting [39]. In warm climates, other *Penicillium* species are present, for instance, *P. citrinum*, which is a common contaminant of rice. In maize and other cereals, several species of *Penicillium* sp. are present during their processing, for example, *P. funiculosum* in preharvest grains only, *P. aurantiogriseum* and *P. viridicatum* in preserved grains, along with *P. citrinum* and *P. oxalicum* which are throughout. Birck and colleagues [40] explored the impact of fungal infection on wheat grain stored for a period of six months. It was reported that 30 days after harvest and storage, the dominant fungus was found to be *Fusarium* spp. However, the numbers reported decreased progressively up to the end of the storage period. After a prolonged storage period of 180 days, wheat samples were 96.7 percent, 46.7 percent, and 80.0 percent contaminated with *Aspergillus*, *Fusarium,* and *Penicillium*, respectively. As a result, there is an immediate demand for suitable methods and strategies to mitigate and/or entirely remove the presence of mycotoxins in cereal grains.

In this regard, the use of plant-based EOs has been suggested as a green alternative to this problem because of their high tolerance in humans and their extensive application in medicine and cooking.

## 3. Bioefficacy of Essential Oils in Protecting Stored Cereal Grains

EOs are natural, aromatic, oily liquids present in most plants produced during secondary metabolism which help in the defense of the plant from insects, pests, microbes, and even environmental conditions [41]. In the search for novel preservatives, EOs may be considered viable substitutes that are environmentally safe and beneficial to both human beings and animals [42]. EOs act as potential preservatives due to their excellent antifungal, antibacterial, and insecticidal properties which are discussed below.

### 3.1. Antifungal Properties of Essential Oils

In 1959, the antifungal effects of EOs were first identified. The particular mechanism that allows EOs to reduce the growth of fungi is still unclear. However, some studies imply that some EOs have both direct and indirect effects on fungal mycelia; they act on the plasma membrane of fungi leading to their disruption, causing cytoplasmic content to leach out and ultimately causing the death of the fungi [43].

Recent studies have investigated the antifungal effects of essential oils on *Aspergillus* and *Fusarium* in cereals. Cinnamon oil demonstrated the most significant fungicidal activity against *A*. *flavus* at a concentration of 0.125%, achieving approximately 85–90% reduction in aflatoxins B_1_ and B_2_. Additionally, cinnamon oil decreased the molecular expression of the *nor-1, afLR, pKsA*, and *afLJ* genes by 94–96% compared to the control. Moreover, Iwayemi et al. [44] assessed the inhibitory effects of six essential oils (cinnamon, clove, eugenol, orange, oregano, and thyme) at varying concentrations (0–0.8 mg/mL) on fungal growth. The antifungal index (AI) for each treatment was measured after 7 days of incubation at 22 °C. Cinnamon oil consistently exhibited the highest AI and the lowest IC_50_ (0.065 mg/mL) against the growth of *Aspergillus* and *Penicillium*, followed by clove oil with an IC_50_ of 0.12 mg/mL. Fusarium spp. was particularly sensitive to cinnamon oil, showing the lowest IC_50_ of 0.006 mg/mL, with eugenol and oregano oil both having IC_50_ values of 0.01 mg/mL.

### 3.2. Antibacterial Properties of Essential Oils

EOs possess remarkable antibacterial properties that are useful in safeguarding stored cereal grains against bacterial infection and rancidity. The antibacterial activity of EOs is associated with the specific structural features of terpenes, phenolics, and aldehydes, which affect the bacterial cell membrane, inhibit biochemical processes, interfere with bacterial respiration, diminish bacterial proliferation on cereal grain surfaces, and interfere with intracellular activities that are crucial for the survival of bacteria [45]. Moreover, the effectiveness of their activity varies across different bacterial strains. Essential oils tend to have a broad range of activity against both Gram-positive and Gram-negative bacteria [46]. In this context, Al-Mahdi et al. [47] reported the antibacterial properties of EOs such as thyme, tea tree, and bergamot, especially for drug-resistant strains such as *Staphylococcus aureus* and *Escherichia coli*. Similarly, Dong et el. [48] revealed the potent inhibitory activity of *Citrus reticulata* essential oil against a wide range of microorganisms, such as *Bacillus subtilis*, *Bacillus pumilus*, and *Candida albicans*, with Minimum Inhibitory Concentrations (MICs) that ranged from 0.04 to 0.32%. In addition, antibacterial properties against *E. coli*, *P. aeruginosa*, and *Klebsiella pneumoniae*, particularly at 1/10 concentrations, were significantly exhibited by essential oils of *Origanum vulgare*, *Lavandula officinalis*, and *Syzygium aromaticum* [49]. Recently, Zhao et al. [50] investigated that among the diverse varieties of essential oil compounds, a 1:1 formulation of cinnamon bark oil and oregano oil provides greater antibacterial activity against *E. coli* and *S. aureus*.

### 3.3. Insecticidal Properties of Essential Oils

The insecticidal efficacy of EOs in preserved cereal grains is an interesting concept, as they can serve as eco-friendly alternatives to synthetic insecticides. Reports have shown that some EOs are quite toxic and repel common pests inhabiting stored grains, thus preserving grains during storage [51]. In this context, research has also demonstrated the larvicidal and antifeeding activities of EOs on development and adult emergence inhibition, egg mortality induction, oviposition inhibition, and influential repellent behaviors [52,53,54,55]. Ankitha et al. [56] suggested that EOs derived from the leaves of *Callistemon lanceolatus* exhibit potential for development as a biopesticide to manage stored product insects, offering a sustainable alternative to traditional chemical insecticides. The findings demonstrated significant insecticidal efficacy against pests including *Tribolium castaneum* and *Callosobruchus maculatus*, with LC_50_ values reflecting substantial contact and fumigation toxicity. Moreover, in their investigation, Wongsawas et al. [57] tested EO from *Plectranthus amboinicus* leaves against *Sitophilus zeamais*, which is a maize weevil. After 144 and 168 h of exposure, EO usage at an air concentration of 2 µL/mL resulted in death of over 90% of maize weevils. At the highest concentration tested (3 µL/mL air), very high mortality rates were observed, as 99–100% maize weevils died from the treatment. The results indicate that *Plectranthus amboinicus* EO could be used as a natural pesticide against maize weevil and could therefore eliminate the use of synthetic insecticides that are harmful to the environment. EOs were used to study the relation of their fumigant activity on a number of the most common pests of stored products beetles: *Sitophilus oryzae*, *Rhyzopertha dominica*, *Tribolium castaneum*, and *Callosobruchus chinensis*. It was observed that all essential oils at a concentration of 0.4% led to the complete death of the insects within twenty-four hours post-application, indicating their high effectiveness without hindering germination in both wheat and chickpea [58]. Kavallieratos et al. [59] reported that EOs obtained from plants belonging to the Apiaceae family such as *Smyrnium olusatrum* and *Trachyspermum ammi* display strong insecticidal properties against a range of stored-product insects, including *Sitophilus oryzae* (L.), *Trogoderma granarium* Everts, *Rhyzopertha dominica* (F.), *Tribolium castaneum* (Herbst), *T. confusum* Jacquelin du Val, *Oryzaephilus surinamensis* (L.), *Alphitobius diaperinus* (Panzer), *Acarus siro* (L.), and *Tenebrio molitor* (L.), in the case of wheat, with full mortality being observed at higher concentrations (1000 ppm).

Therefore, recent advancement in nanotechnology may serve as a novel and efficient strategy to effectively utilize the antimicrobial property of EOs, ensuring the regulated release of essential oils and enhancing their efficacy. Nanotechnology involves the effective encapsulation of essential oils into a carrier system, resulting in nano-range particles (size < 100 nm) that conceal the undesirable aroma of EOs, protect it from oxidative damage, and also enhance their antimicrobial efficacy that enables the development of suitable eco-friendly nano-preservatives with controlled release properties to safeguard food commodities from post-harvest losses [55]. An effective way to raise the caliber, security, and usefulness of food items is to employ nanoparticles as delivery systems for EOs. Thus, one of the fastest-growing industries in recent years is the application of nanotechnology in the food sector [60].

## 4. Nanoencapsulation Process and Its Advantages

Nanoencapsulation can be defined as a technology which involves the packaging of EOs or their bioactive constituents in a wall matrix, leading to the formation of nanocapsules whose size ranges between nanometers to a few millimeters. The nanorange size of particles offers a larger surface area that allows effective dispersion throughout food matrices. Other physico-chemical features, like the shape of nanoparticles, dissolution, encapsulation efficiency (amount of essential oil encapsulated into nanoparticle), etc., vary depending on the method of distribution and the encapsulation technique used for nanocapsule formation [61].

Moreover, nanoencapsulation dramatically improves the antimicrobial capacity of EOs since nanoencapsulation enhances the stability and solubility of EOs and controls their release. This process of encapsulation ensures the protection of EOs using coatings that uphold their bioactivity while at the same time allowing for the effective targeted release to microbial cells [62]. In nanoencapsulation, the choice of wall material is of great importance. Lecithin, gelatin, albumin, and legumin are the most frequently applied wall materials. They are naturally amphipathic and, therefore, act as good emulsifiers. Additionally, proteins (whey protein concentrate and soy protein) are natural, non-toxic, and biodegradable wall materials often used in the agri-food industry for various applications. Bioactive constituents are encapsulated in polysaccharides such as starch, chitosan, dextrin, alginates, and gums [63]. In this context, lipid bilayer vesicles are able to provide an ideal environment wherein lipophilic substances, including the hydrophobic components of essential oils, can be incorporated. Both passive and active loading strategies may be utilized to bring about efficient encapsulation to permit a higher percentage of bioactives to effectively entrap themselves within nanovesicles [64]. Different wall materials have been used to encapsulate various bioactive components to enhance their bioactivity.

Although several strategies for encapsulating substances with biological activity have been investigated, freeze-drying and spray-drying are extensively employed in the food sector. Freeze-drying or lyophilization is a drying process that involves the freezing of the solvent followed by its sublimation, usually performed at −50 to −85 °C temperature and 0.3–1.3 mbar pressure, while the spray-drying process involves drying the nanoemulsion by spraying it into a chamber that contains hot circulating air, leading to the formation of powdered nanocapsules. The emulsification technique is often the initial phase of encapsulation. It involves mixing two immiscible phases, hydrophilic (water) and lipophilic (oil), with the help of an emulsifier that acts as a surface-active agent, resulting in the formation of an emulsion. Two types of emulsification procedures are generally utilized for the production of encapsulation systems, i.e., the top–down and the bottom–up approaches [65]. The top–down approaches entail the transformation of huge structural materials into smaller structures by diminishing their size and reshaping them using external mechanical destructive forces. These techniques typically encompass microchannel homogenizers, microfluidization, and high-pressure homogenization [66]. They are often employed for encapsulating hydrophobic and hydrophilic chemicals; however, they offer little control over the particle size and structure of the resultant emulsion and are appropriate only for a restricted range of matrices. The bottom–up technique usually includes processes such as self-assembly, phase separation, and emulsification, which do not require any mechanical action but utilize the system’s intrinsic chemical energy for encapsulation, which is often influenced by pH, concentration, temperature, and ionic strength [67]. At the same time, low-energy techniques act as pre-steps for other several nanoencapsulation processes, such as complex coacervation, electro-spinning, spray-drying, electro-spraying, and extrusion [68]. They provide an enhanced control over the capsule’s characteristics and require less energy. Nonetheless, low-energy techniques need substantial quantities of stabilizers and are applicable only for a restricted variety of oils and surfactants. Numerous strategies for encapsulation have been suggested in the research; however, none can be deemed standard or universally applicable for all physiologically active substances. The optimal technique may be determined based on the characteristics of the core component and the encapsulating material, encompassing their molecular weight, particle size distribution, solubility, polarity, and food matrix structure [69].

Increased bioavailability, decreased volatility, and increased thermal and chemical stability are all the results of encapsulation, which also preserves the functional characteristics of bioactive components. Since there is more area available per unit volume when the size of the nanoparticle is reduced, the solubility, bioactivity, and delivery efficacy of bioactive components are all enhanced [70]. Research on nanotechnology is still in its infancy. The pharmaceutical industry has been exploring nanotechnology over the last couple of years, and now the food industry is taking a keen interest in it as a means of delivering dietary supplements and preservatives extracted from plants. A number of advantages in the improvement of dispersion; stability against oxidation; safety against oxidative destruction, with little to no negative impact on the organoleptic quality of the relevant food items; ease of handling and solubility; as well as controlled release, and enhanced bioavailability of the substance are provided by the nanoencapsulation of essential oils. Therefore, the application of nanotechnology in designing EO-based preservatives may enhance their efficiency in the food chain [64].

**Figure 1:**
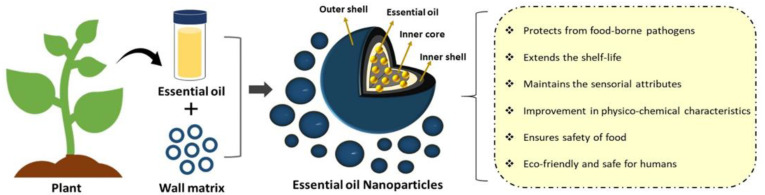
Potential benefits of essential oil nanoparticles in food preservation.

Biocompatible polymeric nanocarriers with EOs packaged in them, irrespective of their morphology, have demonstrated the ability to preserve or enhance the chemical and physical stabilities of EOs and bioactives, thereby mitigating their primary drawbacks and safeguarding them from unintended chemical breakdown under challenging food-processing and environmental conditions. When EOs are encased within the polymer matrix, their biological activity is preserved, but their interactions with dietary ingredients can either be completely excluded or greatly reduced. Since they ensure a more even distribution of lipophilic chemicals in aqueous-based matrices for food, hydrophilic polymers are commonly employed to encapsulate essential oils [71]. In addition, conjugating the EOs with polymeric nanoparticles would provide an extended-release rate inside the food matrix, thus preventing spoiling and extending the shelf life of food items while they are being transported or stored. Hence, nanoencapsulated essential oils have the potential to serve as next-generation green preservatives for the long-term preservation of food commodities [72]. The various advantages of using essential oil nanoparticles in food preservation are shown in Figure 1.

## 5. Application of Nanoencapsulated Essential Oils in Grain Storage

Essential oils are volatile substances that readily degrade at ambient temperature. It is therefore essential to employ various methods or strategies to boost their activity and stability. Khalili et al. [73] employed chitosan-benzoic acid nanogel to enhance the activity, stability, and accessibility of thyme oil. The researchers found that when nanogel-encapsulated thyme oil was used, *Aspergillus flavus* was able to reproduce at a minimum inhibitory concentration of 300 mL/L under sealed conditions, in addition to an increased concentration of 500 mL/L under non-sealed conditions. On the other hand, free thyme essential oil only inhibited the development of these fungi at 400 mL/L and 1000 mL/L under sealed and non-sealed conditions, respectively. When free thyme essential oil molecules were embedded in chitosan-benzoic acid nanogels, they exhibited a strong improvement in half-life along with antifungal activity [74]. Mohammadi and colleagues [75] investigated the encapsulation of Zataria multiflora essential oil (ZEO) into chitosan nanoparticles. The aim of this research was to evaluate the antifungal activity of essential oil against the fungus causing gray mold disease, *Botrytis cinerea*, where efficacy of encapsulation was estimated. Researchers encased essential oil in chitosan nanoparticles that ranged from 125 to 175 nm, employing an ionic gelation technique. Valencia-Sullca et al. [76] used lecithin liposomes for preparing an antimicrobial film of cinnamon essential oil extracted from leaves along with eugenol. They found that liposome-based encasing exhibits an improved water vapor barrier capacity and increased film expansion compared to the unencapsulated compounds. Some examples of nanoencapsulation strategies that have been employed in the preservation of stored cereal grains are discussed in Table 1.

## 6. Hurdles in Application of Nanoencapsulated Essential Oils

Nanoencapsulation improves the stability and efficacy of EOs, facilitates regulated release, and minimizes sensory effects. Despite these benefits, many obstacles restrict the extensive use of nanoencapsulated EOs in the preservation of cereal grains. These problems encompass technological, economic, regulatory, and consumer-related dimensions. Some of the major obstacles associated with the implementation of EO-based nanoformulations include the following:

I. Microbial and pest resistance:

Although EOs have extensive antibacterial and insecticidal properties, not all grain pests and microbial species are uniformly impacted by them. Certain insects or microbial species might develop resistance over time, limiting the efficacy of EOs. Moreover, the use of nanoencapsulation may improve the distribution of EOs, but it does not ensure a consistent response among all species. Therefore, nanoencapsulated EOs may require integration with other preservation methods to avoid the development of resistance [61]. The effectiveness of EOs may differ based on the specific type of grain pest. The diversity in reaction hinders the use of nanoencapsulated EOs as an ideal solution for grain preservation. Achieving comprehensive effectiveness against various pests and grain varieties presents a technological challenge that demands more investigation.

II. Cost and economic limitations:

The process of designing nanoencapsulated EOs incorporates advanced technological methods, such as utilizing nanocarriers prepared from proteins, lipids, or synthetic polymers, which greatly increases the cost of production as compared to conventional means such as artificial preservatives. Economic impediments hinder the utilization of costly apparatus, and this aspect remains a chief obstruction to widespread implementation, especially in poorer regions where grain storage is crucial [115].

III. Scientific and technical challenges:

A significant challenge in the nanoencapsulation of EOs is achieving increased encapsulation efficiency. Different EOs have different volatility and stability profiles, and other nanocarriers are not as good at retaining them. Cereal grains have unique surface properties that affect the adherence and effectiveness of EOs encapsulated in nanoparticles, such as wheat, maize, and rice. Investigations are still focused on creating nanoformulations that coat these grains with uniformity and efficiency, maintaining their stability, and making it easier for their active components to be released over time [116]. A primary advantage of nanoencapsulation is its capacity for the regulated release of active substances. Still, attaining uniform release profiles during prolonged storage durations that can last for months or years is a technological problem. Variations in temperature and humidity inside grain storage facilities can compromise the integrity of nanocarriers, resulting in the premature degradation or unregulated release of EOs [62]. Moisture, light, and temperature may negatively influence nanoencapsulated EO stability. In grain storage facilities, variable environmental conditions might cause nanocarriers to break down prematurely, releasing EOs or destroying their active characteristics [117]. Technical challenges must be overcome to ensure nanoencapsulated essential oils remain effective in real-world storage conditions for large-scale application.

IV. Possible effects on cereal grain quality:

EOs possess potent aromas and flavors that can influence the sensory characteristics of the grains. Although nanoencapsulation might partially mask these attributes, inadequate encapsulation or unpredictable release may still result in unfavorable alterations in flavor, fragrance, or texture [118]. Consumers may exhibit a reduced acceptance of grains preserved with nanoencapsulated EOs if there is a significant impact on the product’s sensory attributes.

V. Regulatory and safety concerns:

The regulatory frameworks for nanotechnology in food applications, such as grain preservation, remain underdeveloped. In several countries, regulatory agencies like the U.S. Food and Drug Administration (FDA) and the European Food Safety Authority (EFSA) have yet to formulate extensive recommendations for the use of nanoparticles in food storage [119]. This lack of clarity discourages enterprises from embracing nanoencapsulated EOs, owing to protracted and complicated regulatory procedures. Concerns regarding the safety and environmental effects of nanoparticles further complicate regulatory approval. One of the major issues is the safety related to the consumption of nanoparticles. Although EOs may be released in a controlled manner through nanoencapsulation, there is still little information available on the long-term health effects of consuming nanoparticles. There is uncertainty over the safety of using these chemicals for food preservation due to their potential for bioaccumulation in humans and the environment. Nanoparticles, because of their small size, can easily enter the human body through ingestion, inhalation, or absorption and interact with cells and tissues, leading to harmful effects such as DNA damage, oxidative stress, and inflammation. Additionally, they might trigger immunological responses in the body that result in allergic reactions or persistent inflammation. Nanoparticles can also interact with the biomolecules responsible for regulating the normal function of the body. This might disturb the regular function and lead to life-threatening toxicities [69]. Hence, it is challenging to secure regulatory approval for the use of nanoencapsulated EOs in grain storage systems, which limits their commercial acceptability.

However, to address issues with cost, permission from regulators, technical obstacles, pest resistance, and environmental sustainability, business leaders, regulators, and customers must work collaboratively to continuously investigate, innovate, and solve problems. In order to fully utilize nanoencapsulated EOs for grain preservation and provide a safe, effective alternative to synthetic preservatives, these obstacles must be overcome.

## 7. Conclusions

The possible microbial threats posed on cereal grains are a cause for concern for the cereal grain industry, owing to the significant effects on the quality and nutritional attributes. There is great potential for the use of EOs as natural preservatives in the protection of cereal grains and other food commodities from microbial contamination and to extend their shelf life. However, the broader use of EOs in various industries is restricted due to its dependence on environmental factors, volatility, and limited solubility. Nevertheless, these limitations can be overcome by nanotechnology, through the use of EOs encased within polymeric matrices which allow for controlled release and targeted delivery. This technology enhances the effectiveness of EOs against microbes while safeguarding cereal grains. With the development of modified structures on molecular size, such as emulsions and nanomaterials, stored grain protection becomes more innovative and practical, ensuring food safety and minimizing the use of synthetic additives. Further, before recommending the use of nanoencapsulated EOs in the food industry, stringent regulatory protocols must be followed, and safety must be ensured to eliminate potential risks that may affect human beings and the environment. Hence, there is a need for integrated, long-term research that focuses on the practical applicability, scalability, sustainability, safety and toxicology, bioefficacy, impact on grain quality, consumer acceptance, and regulatory aspects of the application of nanoencapsulated EOs in cereal grain preservation. Advances in this field will not only aid in the development of efficient and sustainable grain preservation techniques, but also create new avenues for improving food security, quality, and market acceptability.

## Figures and Tables

**Table 1:** Application of nanoencapsulated essential oils in cereal grain preservation.

Cereal Grain/Processed Products	Essential Oil/ Bioactive Compounds	Method of Encapsulation	Polymer Used/ Type of Nanocarrier	Effective Against Microbial Agents/ Insect Pests	Mode of Action of Nanocapsule	References
Maize	*Satureja montana* and *Origanum virens*	Thin-film hydration (TFH) method with homogenization and sonication	Non-ionic surfactant-based lipid vesicles (niosomes)	*Aspergillus flavus* and aflatoxin B_1_	Niosome-encapsulated *S. montana* EO was most effective in controlling AFB_1_ production by *A. flavus*, while both EOs were able to control fungal growth, with maximum reduction up to 79% and 69% for *S. montana* and *O. virens* EOs, respectively, after 45, 60, and 75 days of incubation.	[77]
	*Cymbopogon martinii*	Ionic gelation	Chitosan	*Fusarium graminearum,* deoxynivalenol and zearalenone	At 700 ppm concentration, nanoparticles were able to inhibit the development of fungi and trigger the production of mycotoxin.	[78]
	*Pimenta dioica*	Ionic gelation	Chitosan	*Aspergillus flavus* and aflatoxin B_1_	Nanoparticles were reported to have antifungal and antiaflatoxigenic activities at 1.6 and 1.0 µL/mL, respectively.	[79]
	Orange (*Citrus sinensis*)	Nanoprecipitation	Zein	*Stenocarpella macrospora*	After four days of incubation, the administration of 4% nanoparticles encapsulated with orange essential oil suppressed over 50% of the mycelial growth.	[80]
	Clove oil	Wash out method, ultrasonication	Whey protein	*Fusarium proliferatum*	Minimum fungicidal concentration (MFC) and minimum inhibitory concentration (MIC) of clove oil against *Fusarium proliferatum* were 50 μL and 9 μL, respectively. The produced clove EO nanoemulsion was successful in lowering the load of fumonisin B_1_ and B_2_ and the development of *Fusarium proliferatum*.	[81]
	Clove oil	Solution reduction method	-	*Bipolaris maydis*, *Rhizoctonia solani* f. sp. *sasakii*, *Macrophomina phaseolina*, *Fusarium verticillioides* and *Sclerotium rolfsii*	At a dosage of 500 mg/L, essential oil-grafted copper nanoparticles (EGC) acted as potential fungicide in controlling phytopathogens that infested maize, namely *B. maydis*.	[82]
	Anethole	Ionic gelation	Chitosan	*Aspergillus flavus* (AF-LHP-VS8) and aflatoxin B_1_	At 0.8 and 0.4 μL/mL, respectively, anethole-loaded chitosan nanoemulsion (Ant-eCsNe) reduced growth and AFB_1_ synthesis, making it effective against *A. flavus* (AF-LHP-VS8) and other food-borne molds. Ant-eCsNe enhanced leakage of cellular constituents and inhibited ergosterol in a dose-dependent manner, indicating fungal plasma membrane as the target site.	[83]
	*Pogostemon cablin* (Blanco) Benth.	Ionic gelation	Chitosan	*Aspergillus flavus* and aflatoxin B_1_	Chitosan-loaded *Pogostemon cablin* EO (PCEO-CN) exhibited concentration-dependent broad-spectrum antifungal and antimycotoxigenic activity. In vivo studies revealed that PCEO-CN was able to protect maize grains from *A. flavus* and aflatoxin B_1_ contamination in up to 30 days of storage.	[84]
	*Cinnamomum cassia*	Spray-drying	Gum arabic and maltodextrin	*Penicillium crustosum*, *Alternaria alternata*, and *Aspergillus flavus*	Encapsulated *C. cassia* EO showed potent antifungal activity against *A. alternata*, *A. flavus,* and *P. crustosum*, with MIC of 5%. During in situ experiments using maize flour samples treated with free EO showed high persistence of aroma as compared to encapsulated EO.	[85]
Wheat	*Cuminum cyminum* (L.) and *Lavandula angustifolia* (Mill.)	Emulsion solvent evaporation technique	Polyethersulfone	*Sitophilus granarius* (L.)	The most minimal relative growth rate (RGR) and efficiency of conversion of ingested food (ECI) were observed in adults subjected to *C. cyminum* nanoparticles at a concentration of 20 ppm. The RGR decreased from 0.037 ± 0.003 mg/mg/day in the control group to −0.176 ± 0.01 mg/mg/day, while the ECI percentage diminished from 16.8 ± 0.99 to −336.31 ± 6.95. It was noted that both essential oils and their nanoparticles exhibited a behavioral influence on *Sitophilus granarius* (L.), and the encapsulation process resulted in enhanced post-ingestive toxicity in the treated adults.	[86]
	*Melissa officinalis* L.	Ionic gelation	Chitosan	*Tribolium castaneum* Herbst	The nanoencapsulated *Melissa officinalis* L. essential oil demonstrated improved insecticidal efficacy as a fumigant, with a sub-lethal concentration (LC_50_) of 0.048 μL/mL air. Additionally, during in situ trial in wheat flour nanoparticles, a notable 50% anti-feedant activity was observed at an effective concentration (EC_50_) of 0.043 μL/mL.	[87]
	*Mentha* X *piperita* (L.)	Ionic gelation	Chitosan	*Tribolium castaneum* (Herbst) *and Sitophilus oryzae* (L.)	Toxicity experiments demonstrated that *M. piperita*-loaded chitosan nanoemulsion exhibited significantly greater efficacy against both stored product pests compared to the control group. The inhibition percentage of Acetylcholinesterase (AChE) activity differed between *S. oryzae* (52.43% and 37.71%) and *T. castaneum* (37.80% and 31.29%) during in vivo trials.	[88]
	Coriander, oil, janesville, caraway, and black seed	Polymerization technology	Urea and formaldehyde	Confused flour beetle *Tribolium confusum* (Jacquelin) and red flour beetle *Tribolium castaneum* (Herbst)	The results showed that *T. confusum* and *T. castaneum* larval mortality increased in a dose-dependent manner. Compared to *T. castaneum* larvae, *T. confusum* larvae responded better to the treatments. Compared to coriander or black seed oil, nanoformulated Janesville oil was more effective. Under storage conditions, nano-formulations of coriander, black seed, or Janesville oils reduce fecundity and adult emergence percentage more than controls or free oils.	[89]
	*Hazomalania voyronii*	High-pressure homogenization	-	*Tribolium confusum* (Jacquelin du Val), *Tribolium castaneum* (Herbst), and *Tenebrio molitor* L.	After seven days of exposure, *T. confusum* (92.1%), *T. castaneum* larvae (97.4%), and *T. molitor* adults (100.0%) died when exposed to 1000 ppm of *Hazomalania voyronii* essential oil nanoemulsion.	[90]
	*Syzygium aromaticum*	Melt-dispersion method	Polyethylene glycol	*Rhyzopertha dominica*	After 72 h of exposure, the toxicity study showed that *Syzygium aromaticum* (clove) essential oil-loaded nanocapsules were efficient against *R. dominica*. The LC_50_ values for free clove oil and nanocapsules were 576.85 ppm and 175.50 ppm, respectively. According to the toxicity index, free oil was 70% less dangerous than nanocapsules. Nutritional indices like relative growth rate, food intake rate, and ingested food conversion efficiency were all reduced by nanocapsules.	[91]
	*Eucalyptus globulus* Labill *and Zataria multiflora* Bioss	Ionic gelation	Maltodextrin and Angum gum	*Ephestia kuehniella* Zeller	The nanoencapsulation of *E. globulus* and *Z. multiflora* EOs enhanced the toxicity effect against *Ephestia kuehniella* by 10.74 and 4.33 times, respectively.	[92]
	*Pelargonium graveolens*	High-energy ultrasonication	-	*Sitophilus oryzae* L.	*Pelargonium graveolens* nanoemulsion showed higher toxicity against *S. oryzae* (LC_50_ value 2.298 ppm/cm^2^) compared to free oil (LC_50_ value 67.66 ppm/cm^2^). When *S. oryzae* adults were exposed to treated wheat grains, their mortality rate increased with an increase in concentration and exposure intervals. At 200 ppm, the nanoemulsion showed potent insecticidal activity, prevented the emergence of *S. oryzae* and protected wheat grains for 3 months.	[93]
	Geranium and bergamot	Melt-dispersion method	Polyethylene glycol	*Tribolium castaneum* (Herbst) and *Rhizopertha dominica* (Fab)	Due to the slow and continuous release of active terpenes, the EO nanoparticles significantly increased the residual contact toxicity. Furthermore, the nanoformulation changed the nutritional physiology of both stored product pests and increased the contact toxicity of EOs.	[94]
	*Achillea biebersteinii*, *A. santolina* and *A. mellifolium*	High-pressure homogenization	-	*Tribolium castaneum* (Herbst)	Of the 3 EOs, *A. biebersteinii* EO showed the highest insecticidal activity, and the adult stage was more vulnerable than larvae. Following 96 h of larval exposure, the LC_50_ values of *A. biebersteinii*, *A. santolina*, and *A. mellifolium*, EOs applied topically were found to be 30.3, 47.8, and 62.3 g/mg insect. When applied as nano-emulsions, the toxicity of all EOs rose significantly, with LC_50_ values roughly four or three times lower than their typical fumigant activity.	[95]
Rice	*Apium graveolens*	Ionic gelation	Chitosan	*Fusarium verticillioides* and fumonisins (FB_1_ and FB_2_)	At 0.8 μL/mL, chitosan nanoparticles loaded with *Apium graveolens* completely inhibited fungal growth, and at 0.8 and 0.6 μL/mL concentrations, they reduced the formation of FB_1_ and FB_2_, respectively.	[42]
	*Pimpinella anisum* and *Coriandrum sativum*	Ionic gelation	Chitosan	*Aspergillus flavus* and aflatoxin B_1_	A binary synergistic formulation of *Pimpinella anisum* and *Coriandrum sativum* (0.75:0.25) essential oils encapsulated in chitosan biopolymer showed promising antifungal and AFB_1_ inhibitory activities at 0.06 and 0.05 μL/mL, respectively.	[96]
	Thyme	Microfluidization	-	*Fusarium graminearum* 10-124-1 and *F. graminearum* 10-125-1	Thyme oil nanoemulsion showed better antifungal activity against mycelium growth for both selected isolates at 7.61 ± 0.09 mg/g and 7.25 ± 0.43 mg/g, respectively.	[97]
	*Thymus vulgaris*-*Origanum compactum*, *Thymus vulgaris*-*Melaleuca alternifolia* and *Thymus vulgaris*-*Mentha piperita*	High-speed homogenization and microfluidization	Chitosan reinforced with cellulose nanocrystals (CH/CNCs)	*Aspergillus niger*, *Aspergillus flavus*, *Aspergillus parasiticus*, and *Penicillium chrysogenum*	For *Aspergillus niger, Aspergillus flavus*, *Aspergillus parasiticus*, and *Penicillium chrysogenum*, CH/CNCs nanocomposite films loaded with all three EO combinations have shown strong antifungal efficacy, preventing their development by 51–77%. In situ tests revealed that the combination of *Thymus vulgaris*-*Origanum compactum* EO (0.19% w/w) nanocomposite treatment and gamma radiation exposure was most successful; the rice maintained its high level of acceptability after cooking for two months without changing its organoleptic characteristics.	[98]
	Hop (*Humulus lupulus* L.)	High-pressure homogenization	-	*Fusarium graminearum*, deoxynivalenol	By administering 750 μg of Hop EO per gram of rice, the nanoemulsion was able to decrease the formation of deoxynivalenol (DON) and its derivatives in rice as well as impede the mycelial development and spore germination of *F. graminearum.* The nanoemulsion altered the total lipid content and chitin in the outer cell membrane, leading to damage in the plasma membrane.	[99]
	*Cinnamomum zeylanicum* Blume	Nanoprecipitation	Cinnamon oil encapsulated with silica nanoparticles	*Corcyra cephalonica* (Stainton)	When *C. cephalonica* larvae were fed mesoporous silica nanoparticles orally, the highest effective toxicity was seen. After six days of exposure, cinnamon oil encapsulated with silica nanoparticles at dosages of 15, 30, 60, and 90 mg was the second most effective therapy, resulting in 16.7, 36.7, 50, and 53.3% mortality, respectively.	[100]
	*Pinus roxburghii* Sarg, *Juniperus communis* L., and *Cupressus sempervirens* L.	Ionic gelation	Chitosan	*Aspergillus flavus* and aflatoxin B_1_	A synergistic formulation was developed by mixing 3 EOs (PJC) encapsulated into chitosan nanomatrix (Nm-PJC). Nm-PJC showed improved antifungal (4.0 µL/mL), antiaflatoxigenic (3.5 µL/mL), and antioxidant activities. In situ trials revealed its effectiveness in protecting rice against lipid peroxidation, fatty acid biodeterioration, and preservation of minerals and macronutrients without compromising organoleptic qualities.	[101]
	*Artemisia annua* L.	-	Chitosan/TPP (tripolyphosphate) and zeolite	*Sitophilus oryzae* L.	Encapsulated A. *annua* EO showed effective insecticidal activity. On treatment with nanocapsules, glutathione S-transferase activity was enhanced while acetylcholinesterase and esterase activity were markedly reduced in the treatment set as compared to the control.	[102]
	Garlic	Melt-dispersion method	Polyethylene glycol	*Tribolium castaneum* (Herbst)	Garlic EO-loaded nanoparticles were highly effective against *T. castaneum* adults. The control efficacy of nanoparticles was over 80% after five months of storage as compared to free oil (11% efficacy) at similar concentration of 640 mg/kg.	[103]
Millets	*Ocimum americanum*	Ionic gelation	Chitosan	*Aspergillus flavus* and aflatoxin B_1_	Nanoemulsion containing chitosan-infused *Ocimum americanum* EO (OAEO-CsNe) showed enhanced antifungal and antiaflatoxigenic activity against *A. flavus* (MIC and MAIC values, recorded as 0.200 and 0.175 µL/mL, respectively. OAEO-CsNe was able to protect stored *Setaria italica* seed samples from AFB_1_ contamination and lipid peroxidation without interfering with the sensorial properties of millets during one year of storage.	[104]
	*Cinnamomum tamala*	Ionic gelation	Chitosan	*Aspergillus flavus* and aflatoxin B_1_	At concentrations of 1.0 and 0.8 μL/mL, respectively, *Cinnamonomum tamala*-loaded chitosan nanoemulsion (CTEO-CsNe) has shown remarkable efficacy in inhibiting the development of *A. flavus* and AFB_1_. The nano-ranged particles were able to interact efficiently with fungal plasma membrane leading to damage and loss of cellular functions. Without changing the organoleptic characteristics, CTEO-CsNe served as a new green preservative protecting *Setaria italica* against lipid peroxidation and fungal degradation during post-harvest storage.	[105]
	Thymol (T), methyl cinnamate (M), and linalool (L)	Ultrasonication and precipitation	Chitosan	*Aspergillus flavus* and aflatoxin B_1_	Nanogel (Ne-TML), a synergistic formulation of TML (1:1:1), protected *Pennisetum glaucum* L. seeds against fungal contamination (75.40%) and aflatoxin B_1_ production (100%) at a concentration of 0.3 μL/mL for six months of storage. The potential antifungal mode of action of Ne-TML was connected to the reduction in ergosterol levels, cellular ion leakage, impairment in carbon-source utilization, mitochondrial functioning, anti-oxidative defense system, and Ver-1 gene of aflatoxin B_1_ synthesis.	[106]
	*Aniba rosaeodora*	Ionic gelation	Chitosan	*Aspergillus flavus* and aflatoxin B_1_	*A. rosaeodora* EO-loaded chitosan nanoemulsion (AREO-CsNe) completely inhibited the growth of *A. flavus* (AFLHPSi-1) and AFB_1_ production at 0.8 and 0.6 μL/mL concentrations, respectively. During a 1 year of storage period, AREO-CsNe completely protected *Setaria italica* seeds from lipid peroxidation and AFB_1_ contamination without affecting their sensory qualities. It also demonstrated an excellent safety profile, with an LD_50_ value of 9538.742 μL/kg body weight.	[107]
Other cereals	Garlic	Ionic gelation	Chitosan	*Aspergillus versicolor*, *A. niger*, and *Fusarium oxysporum*	Garlic EO-containing nanoparticle compositions showed strong antifungal action against *Fusarium oxysporum*, *A. versicolor*, and *A. niger*. Additionally, they increased the fresh weight of barley, oats, and wheat as well as their emergence, root, and shoot development.	[108]
Bread	Oregano (*Origanum vulgare* Linneus) and thyme (*Thymus vulgaris*)	Nanoprecipitation method	Zein	*Listeria monocytogenes* ATCC 7644, *Staphylococcus aureus* ATCC 2593, *Escherichia coli* ATCC 25922, *Salmonella enterica* serovar Typhimurium ATCC 14028	It was shown that oregano and thyme essential oil-loaded nanocapsules had a greater antibacterial activity against Gram-positive bacteria as opposed to Gram-negative bacteria. These nanocapsules increased the shelf life of bread for 21 days without producing any colonies of mold or yeast and remained thermally stable throughout the baking process.	[109]
	Clove bud (*Syzygium aromaticum*) and oregano (*Origanum vulgare*)	Low-speed mixing and ultrasonication	Methylcellulose	*Aspergillus niger* (ATCC 16404) and *Penicillium* sp. (ATCC 2147)	During a 15-day storage period, both essential oil-loaded nanodroplets decreased the amounts of mold and yeast in sliced bread; the antibacterial qualities of the nanodroplets were further enhanced due to their small size.	[110]
	Carvone	Ionic gelation	Chitosan	*Aspergillus flavus* and aflatoxin B_1_	At 0.5 and 0.4 µL/mL concentration, respectively, nanoencapsulated carvone effectively suppressed *A. flavus* growth and AFB_1_ production. In situ research revealed that nanoencapsulated carvone film was successful in regulating the production of *A. flavus* and AFB_1_ in sliced bread over a 15-day storage period at 25 ± 2 °C and 75% relative humidity. It also preserved the CO_2_ and O_2_ compositions in sliced bread without altering its organoleptic characteristics.	[111]
	Lemongrass (*Elionurus* sp.)	Electrospinning	Cassava starch	*Penicillium crustosum* and *Aspergillus flavus*	Cassava starch–lemongrass EO (LEO) fibers were investigated for their ability to inhibit *Penicillium crustosum* and *Aspergillus flavus*. When compared to alternative treatments, 40% LEO fibres demonstrated strong antifungal activity for both systems in in situ studies (one applied directly to the bread dough and the other as a membrane in active bread packaging), lowering the fungal count.	[112]
Barley malt	*Litsea cubeba*	Homogenization and ultrasonication	Chitosan	Deoxynivalenol	In addition to lowering the build-up of deoxynivalenol during malting, the addition of *Litsea cubeba* essential oil-loaded chitosan-based secondary emulsion enhanced the malt’s quality.	[113]
Rice flour	Curry plant	Homogenization	Liposomes	*Bacillus cereus* ATCC 14579	Strong antibacterial action against *B. cereus* was demonstrated by curry plant EO (MIC = 0.5 mg/mL). Following two, three, and four days of liposome treatment at 20 °C, the population of B. cereus in rice flour decreased by 4.64, 5.05, and 5.90 logs, respectively. Curry plant EO-containing liposomes may work by increasing the permeability of the cell membrane, which allows intracellular chemicals to seep out and inhibit *B. cereus*.	[114]

## Data Availability

No new data were created or analyzed in this study. Data sharing is not applicable to this article.

## Abbreviations

AChE	Acetylcholinesterase
AFB_1_	Aflatoxin B_1_
AFB_2_	Aflatoxin B_2_
AFM_1_	Aflatoxin M_1_
AI	Antifungal Index
DON	Deoxynivalenol
EC_50_	Effective Concentration 50
ECI	Efficiency of Conversion of Ingested Food
EFSA	European Food Safety Authority
EGC	Essential oil-Grafted Copper nanoparticles
EOs	Essential Oils
FB_1_	Fumonisin B_1_
FB_2_	Fumonisin B_2_
FDA	US Food and Drug Administration
GRAS	Generally Recognized as Safe
IC_50_	Half-maximal Inhibitory Concentration
LC_50_	Lethal Concentration 50
MAIC	Minimum Aflatoxin Inhibitory Concentration
MBC	Minimum Bactericidal Concentration
MFC	Minimum Fungicidal Concentration
MIC	Minimum Inhibitory Concentration
OTA	Ochratoxin A
RGR	Relative Growth Rate
TFH	Thin-film hydration
